# Quantum Phase Transition as a Promising Route to Enhance the Critical Current in Kagome Superconductor CsV_3_Sb_5_

**DOI:** 10.1002/advs.202410099

**Published:** 2024-10-16

**Authors:** Wenyan Wang, Lingfei Wang, Xinyou Liu, Chun Wai Tsang, Zheyu Wang, Tsz Fung Poon, Shanmin Wang, Kwing To Lai, Wei Zhang, Jeffery L. Tallon, Swee K. Goh

**Affiliations:** ^1^ Department of Physics The Chinese University of Hong Kong Shatin Hong Kong China; ^2^ Department of Physics Southern University of Science and Technology Shenzhen Guangdong 518055 China; ^3^ Robinson Institute Victoria University of Wellington Wellington 6140 New Zealand

**Keywords:** critical current, high‐pressure, kagome superconductors, quantum phase transition

## Abstract

Developing strategies to systematically increase the critical current, the threshold current below which the superconductivity exists, is an important goal of materials science. Here, the concept of quantum phase transition is employed to enhance the critical current of a kagome superconductor CsV_3_Sb_5_, which exhibits a charge density wave (CDW) and superconductivity that are both affected by hydrostatic pressure. As the CDW phase is rapidly suppressed under pressure, a large enhancement in the self‐field critical current (*I*_c, sf_) is recorded. The observation of a peak‐like enhancement of *I*_c, sf_ at the zero‐temperature limit (*I*_c, sf_(0)) centered at p* ≈ 20 kbar, the same pressure where the CDW phase transition vanishes, further provides strong evidence of a zero‐temperature quantum anomaly in this class of pressure‐tuned superconductor. Such a peak in *I*_c, sf_(0) resembles the findings in other well‐established quantum‐critical superconductors, hinting at the presence of enhanced quantum fluctuations associated with the CDW phase in CsV_3_Sb_5_.

## Graphical abstract

This study demonstrates that the self‐field critical current of the kagome superconductor CsV_3_Sb_5_ is significantly enhanced by at least 10‐fold when CsV_3_Sb_5_ is driven through a quantum phase transition at ≈20 kbar. This peak suggests enhanced quantum fluctuations linked to the destabilization of the charge density wave phase in CsV_3_Sb_5_, paving the way for investigating similar effects in other material families.

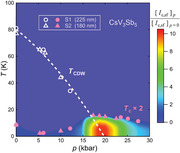

## 1 Introduction

Quantum phase transition, a phase transition at absolute zero temperature triggered by a non‐thermal tuning parameter, can stabilize exotic phases such as superconductivity.^[^ ^1^, ^2^ ^]^ Across a wide variety of material systems, a dome‐shaped superconducting phase is often observed when a neighboring phase is suppressed by non‐thermal parameters such as pressure, strain or chemical doping. Interestingly, the maximum superconducting transition temperature *T*_c_ is often found near the zero‐temperature phase boundary, suggesting that the robustness of the condensate, which is essential for practical applications, is inherently linked to the physics associated with the quantum phase transition. In addition to *T*_c_, the current‐carrying capability of a superconductor is fundamentally important and the critical current (*I*_c_) is another parameter to optimize. Thus, it is crucial to explore if quantum phase transition can also play a positive role in enhancing *I*_c_.

The recently discovered family of kagome superconductors AV_3_Sb_5_ (A = K, Rb, Cs) undergoes a charge density wave (CDW) transition at ≈100 K, and a superconducting transition below 10 K.^[^ ^3^, ^4^, ^5^, ^6^, ^7^, ^8^, ^9^, ^10^, ^11^, ^12^, ^13^, ^14^, ^15^, ^16^, ^17^, ^18^, ^19^, ^20^, ^21^, ^22^, ^23^, ^24^ ^]^ Particularly in CsV_3_Sb_5_, it has been pointed out that the hybridization between V and Sb orbitals plays a critical role in mediating the CDW phase transition.^[^ ^23^ ^]^ The CDW phase itself is complicated, with different charge order patterns reported.^[^ ^24^, ^25^, ^26^, ^27^ ^]^ Under pressure, the CDW can be suppressed and, for CsV_3_Sb_5_, *T*_c_ shows a peculiar double‐dome behavior.^[^ ^6^, ^14^ ^]^ Interestingly, the second dome in CsV_3_Sb_5_ is centered around the extrapolated critical pressure p* where the CDW disappears. Thus, the temperature–pressure (*T*–*p*) phase diagram of CsV_3_Sb_5_ resembles the phase diagram of many quantum‐critical superconductors. The possibility of a quantum critical point (QCP) at p* has been put forward theoretically.^[^ ^28^, ^29^ ^]^ On the experimental front, high‐pressure nuclear quadrupole resonance reported a diverging resonance linewidth at p*,^[^ ^30^ ^]^ and the initial slope of the upper critical field, $-{\left(dH_{c2}/dT\right)}_{T_{c}}$, was found to peak at p*.^[^ ^6^ ^]^ Recently, high‐pressure Shubnikov‐de Haas oscillations detected a noticeable enhancement of the quasiparticle effective masses on approaching p*.^[^ ^31^ ^]^ All these observations suggest the presence of quantum fluctuations that can mediate superconducting pairing. Hence, CsV_3_Sb_5_ is an ideal system to investigate if quantum phase transition can drive the enhancement of *I*_c_.

In this work, we measure the self‐field transport critical current (*I*_c, sf_), i.e. transport critical current without applying an external magnetic field, over a wide pressure range straddling across p*. We observe a drastic enhancement of the critical current when the same set of samples was tuned by pressure. The use of the same samples eliminates extrinsic factors such as the uncertainty in the geometric factor or random potentials due to impurities. In particular, the zero temperature limit of *I*_c, sf_, denoted as *I*_c, sf_(0), of CsV_3_Sb_5_ peaks sharply at p*. This observation further confirms the occurrence of a quantum phase transition beneath the superconducting dome. Together with existing experimental data from other probes, our work establishes access to a quantum phase transition as a promising route to optimize *I*_c_. Finally, we show that the measurement of *I*_c, sf_ provides a systematic means to extract key superconducting parameters of CsV_3_Sb_5_ under pressure.

## 2 Results and Discussion

### 2.1 Construction of *T*–*p* Phase Diagram

**Figure 1:**
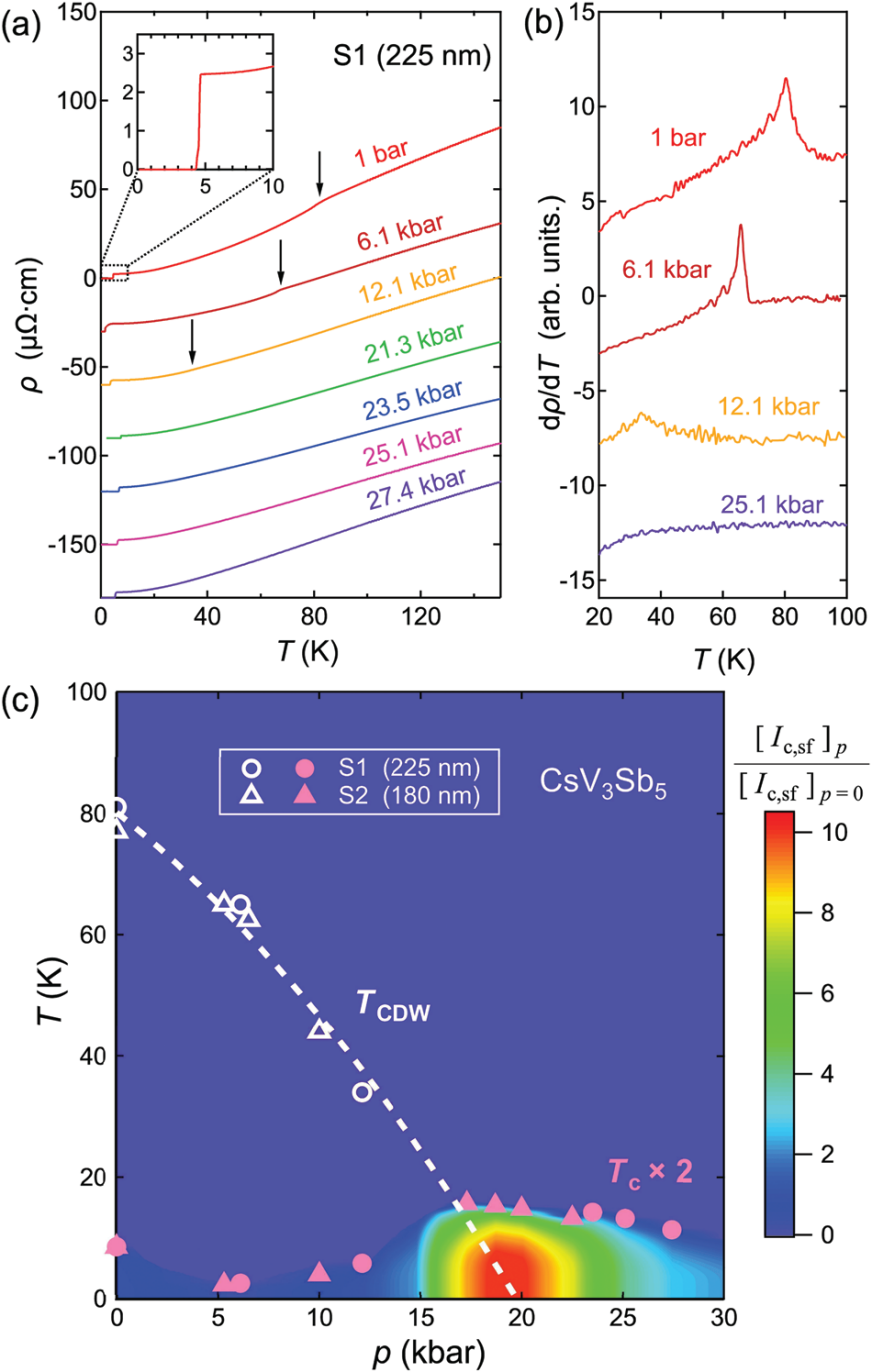
a) Temperature dependence of the electrical resistivity ρ at various pressures in CsV_3_Sb_5_ (S1). All of the traces are measured upon warming up. For clarity, all traces, except the dataset at 1 bar, are successively down‐shifted by 30 µΩ ·cm. The black arrows indicate the *T*_CDW_ and the inset displays the data near the superconducting transition. b) Temperature dependence of *d*ρ/*dT* in S1 at four typical pressures. *T*_CDW_ is determined as the temperature at which *d*ρ/*dT* peaks. c) *T*–*p* phase diagram of CsV_3_Sb_5_ thin flakes with a contour map of the normalized *I*_c, sf_ for S1 and S2 overlaid. See the main text for details. White circles and triangles are *T*_CDW_ of S1 and S2 at various pressures. The white dashed curve indicates the implied, and extrapolated border of CDW phase. The CDW phase is gradually suppressed under pressure and ultimately vanishes at p* ≈ 20 kbar. Pink circles and triangles are *T*_c_ of S1 and S2 at various pressures, demonstrating a double‐dome pressure dependence. *T*_*c*_ is determined by the temperature at which the resistivity reaches zero.

**Figure** **1**a displays the temperature dependence of resistivity for CsV_3_Sb_5_ (S1), which has a thickness of 225 nm, at various pressures. At ambient pressure, a kink is visible at *T*_CDW_ = 80 K, corresponding to the CDW transition well‐documented in the literature for thin CsV_3_Sb_5_ flakes.^[^ ^32^, ^33^, ^34^, ^35^, ^36^, ^37^ ^]^ The transport signature associated with the CDW transition can be best visualized in the thermal derivative *d*ρ/*dT* (Figure 1b). At a lower temperature, the electrical resistivity vanishes at *T*_c_ = 4.3 K (see inset of Figure 1a), signifying a superconducting transition, consistent with previous reports on thin flakes of CsV_3_Sb_5_.^[^ ^32^, ^33^, ^37^ ^]^ The higher *T*_c_ in CsV_3_Sb_5_ thin flakes could be attributed to the biaxial strain effect, which is unavoidable in device architecture (Figure S5, Supporting Information).

Under pressure, the anomaly associated with the CDW transition is weakened. To track *T*_CDW_, we follow the peak in *d*ρ/*dT*. At 12.1 kbar, *T*_CDW_ decreases to 34 K. Concomitantly, *T*_c_ first decreases under pressure then begins to increase. The data for another flake (CsV_3_Sb_5_ (S2)) show a similar pressure dependence. Combining all data from S1 and S2, a *T*–*p* phase diagram can be constructed (Figure 1c), which clearly shows that *T*_c_ peaks around the pressure p* where *T*_CDW_ extrapolates to 0 K. Unlike the *T*–*p* phase diagram of the bulk sample, the maximum of the lower‐pressure dome shifts from ≈7 kbar to or even below ambient pressure in our flake, while the high‐pressure dome remains centered at ≈20 kbar. Our *T*–*p* phase diagram is similar to that constructed by Ye et al.^[^ ^37^ ^]^ As a preview, we overlay the temperature dependence of *I*_c, sf_ at various pressures as a contour map on the *T*–*p* phase diagram. We can see that *I*_c, sf_ also experiences a drastic enhancement near p*. In the remaining texts, we will demonstrate the construction of this contour map.

### 2.2 *I*_c, sf_ as a Probe of the Superconducting Gap

**Figure 2:**
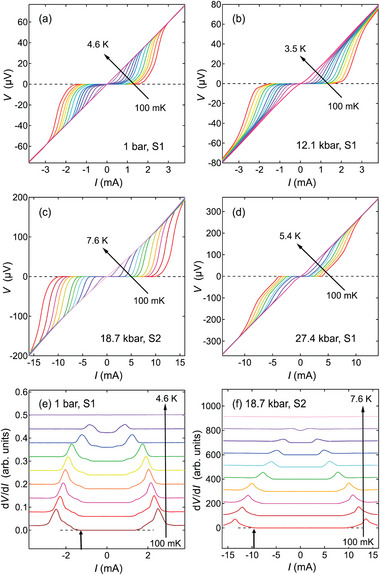
Temperature dependence of *V*–*I* relations at a) ambient pressure, b) 12.1 kbar, c) 18.7 kbar, and d) 27.4 kbar in S1 (225 nm) and S2 (180 nm). The solid arrows indicate the increase of temperature from 100 mK to just above *T*_c_. The calculated first derivative of *V*(*I*), *dV*/*dI* of e) S1 at ambient pressure and f) S2 at 18.7 kbar. The short arrows indicate the value of self‐field critical current, which is defined when *dV*/*dI* first deviates from zero. The long arrows demonstrate the increase of temperature from 100 mK to the temperature just above *T*_c_. All *V*–*I* curves and their derivatives are shown in Figures S2 and S3 (Supporting Information).

Based on the *T*‐*p* phase diagram constructed, we measure a series of voltage–current (*V*–*I*) curves in the superconducting state at various pressures. Representative data are shown in **Figure** **2**a–d. Above *T*_c_ (e.g. 4.6 K at 1 bar), *V* is linear in *I*, showing a typical ohmic behavior of a metal. At the lowest temperature for each pressure (e.g., 100 mK at 1 bar), *V* is zero for low current but increases drastically when the current reaches a threshold. This is a typical critical current behavior. The *V*–*I* curves for the intermediate temperatures evolve systematically between the two limits.

Following the same practice as our earlier work,^[^ ^33^ ^]^ we define *I*_c, sf_ as the current at which *dV*/*dI* deviates from zero. Representative *dV*/*dI* curves are displayed in Figure 2e,f, with the short vertical arrows denoting *I*_c, sf_ at 100 mK for 1 bar and 18.7 kbar, respectively. The *dV*/*dI* curves clearly demonstrate the smooth evolution of *I*_c, sf_ as a function of temperature. We also note the different current scales in Figure 2e,f. Therefore, *I*_c, sf_ is roughly an order of magnitude larger at 18.7 kbar than that at 1 bar.

**Figure 3:**
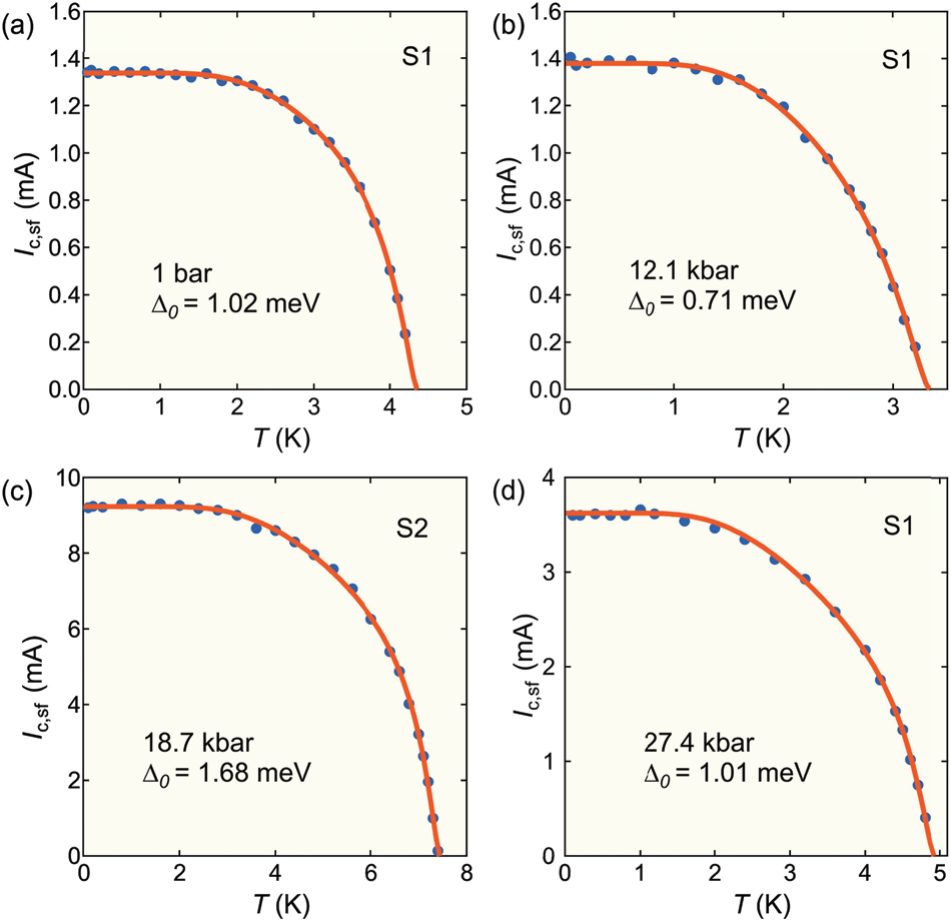
Temperature dependence of *I*_c, sf_ at four representative pressures. The solid curves are the single *s*‐wave gap fits. The extracted superconducting gap Δ_0_ are 1.02 meV at ambient pressure, 0.71 meV at 12.1 kbar, 1.68 meV at 18.7 kbar and 1.01 meV at 27.4 kbar, respectively. *I*_c, sf_(*T*) at all pressures are shown in Figure S4 (Supporting Information).

To have a detailed examination of the pressure dependence of *I*_c, sf_(*T*), the extracted *I*_c, sf_(*T*) from Figure 2 are plotted in **Figure** **3** at various pressures. When the temperature decreases from *T*_c_, *I*_c, sf_(*T*) at first rises rapidly and then saturates when the temperature is sufficiently low. *I*_c, sf_(*T*) curves at all pressures are qualitatively similar. Quantitative information can be obtained by noting the physical significance of *I*_c, sf_. A series of studies^[^ ^33^, ^38^, ^39^, ^40^, ^41^ ^]^ have shown that the transport critical current density at zero magnetic field, *J*_c, sf_, is related to the superfluid density ρ_s_∝λ^−2^, where λ denotes the penetration depth. Specifically, when the half thickness *b* of the flake is less than λ, *J*_c, sf_ is given by Refs. [38, 39]

(1) $$J_{c,\mathrm{sf}}=\frac{\phi_{0}}{4\pi \mu_{0}\lambda^{3}}\left(\ln\left(\frac{\lambda }{\xi }\right)+0.5\right)$$

where ϕ_0_ is the flux quantum, µ_0_ is the vacuum permeability and ξ is the coherence length.

The thickness 2*b* of S1 and S2 are 225 nm and 180 nm, respectively. Furthermore, the penetration depth at the 0 K limit, λ(0), is ≈450 nm.^[^ ^42^ ^]^ Thus, the thin‐limit Equation (1) is appropriate here. The temperature dependence of *J*_c, sf_ is dominated by the temperature dependence of λ, with a reasonable assumption that the temperature dependence of λ/ξ under the logarithm can be disregarded. Consequently, *J*_c, sf_(*T*)/*J*_c, sf_(0) directly reflects [λ^−2^(*T*)/λ^−2^(0)]^3/2^, which is influenced by the magnitude of the superconducting gap. From the NMR measurements, a Hebel–Slichter coherence peak just below *T*_c_ suggests CsV_3_Sb_5_ exhibits an *s*‐wave superconductivity.^[^ ^43^ ^]^ Moreover, both the muon spin rotation and the tunnel diode oscillator results of magnetic penetration depth indicate that the superconducting order parameter has a two‐gap *s*‐wave symmetry, indicating nodeless superconductivity in CsV_3_Sb_5_.^[^ ^42^, ^44^, ^45^, ^46^ ^]^ Furthermore, angle‐resolved photoemission spectroscopy measurements on CsV_3_Sb_5_, tuned by isovalent Nb/Ta substitutions of V, also suggest a nodeless electron pairing.^[^ ^47^ ^]^ Finally, the previous self‐field critical current study by some of us also demonstrates nodeless superconductivity in CsV_3_Sb_5_, regardless of the presence of time reversal symmetry.^[^ ^33^ ^]^ Therefore, we attempt to fit the low‐temperature *J*_c, sf_(*T*) by taking an *s*‐wave gap, where λ^−2^(*T*)/λ^−2^(0) is given by^[^ ^48^ ^]^:

(2) $$\frac{\rho_{s}\left(T\right)}{\rho_{s}\left(0\right)}=\frac{\lambda^{-2}\left(T\right)}{\lambda^{-2}\left(0\right)}=1-2\sqrt{\frac{\pi \Delta_{0}}{k_{B}T}}\exp\left(-\frac{\Delta_{0}}{k_{B}T}\right)$$

**Figure 4:**
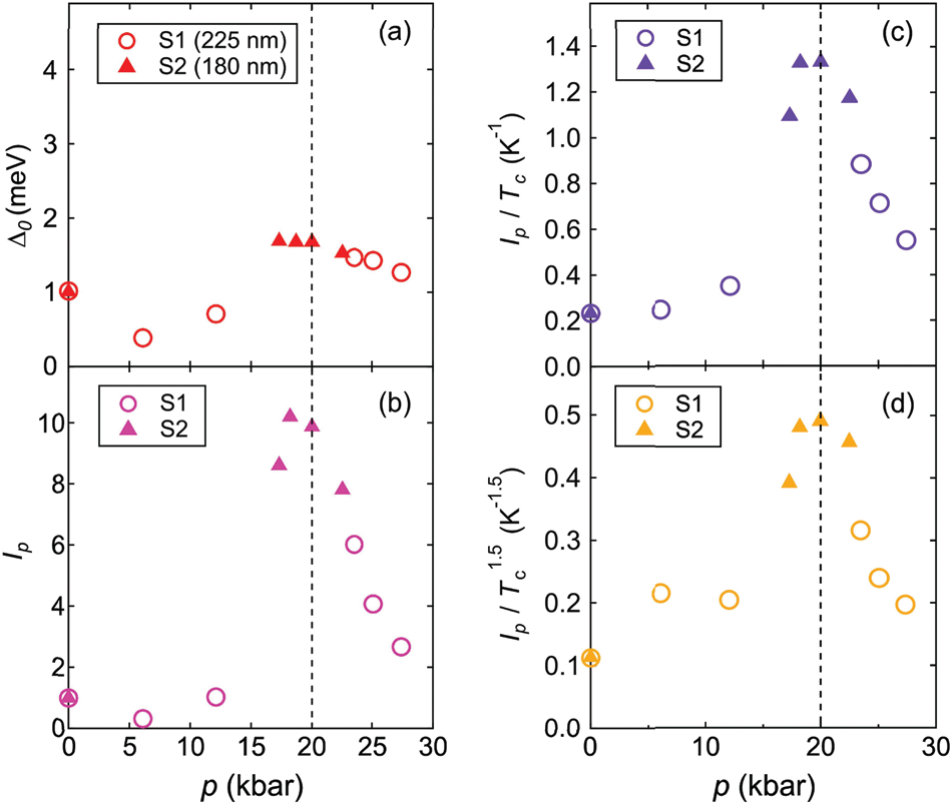
Pressure dependence of a) the superconducting gap Δ_0_, b) *I*_*p*_ = [*I*_c, sf_(0)]_*p*_/[*I*_c, sf_(0)]_*p* = 0_, c) *I*_*p*_/*T*_c_, (d) $I_{p}/T_{c}^{1.5}$. The open circles and solid triangles in (a–d) denote S1 and S2, respectively. Both S1 and S2 have a thickness ≈200 nm. In (a–d), p* is denoted by the vertical dashed line.

As shown in Figure 3, the combination of Equations (1) and (2) can describe the experimental data very well, even at higher temperatures. From these analyses, the pressure dependence of the superconducting gap Δ_0_ can be extracted. At ambient pressure, the superconducting gap Δ_0_ = 1.02 meV (2.75 *k*_B_ *T*_c_), which is larger than the BCS weak coupling limit and consistent with the previous results 0.95 meV (2.70 *k*_B_ *T*_c_) and 1.08 meV (2.84 *k*_B_ *T*_c_).^[^ ^33^ ^]^ At higher pressures, Δ_0_ varies in a non‐monotonic manner (**Figure** **4**a) but appears to track the pressure dependence of *T*_c_. However, the gap‐to‐*T*_c_ ratio shows a noticeable pressure dependence – 2Δ_0_/*k*_B_ *T*_c_ ranges from 4.8 to 6.9 throughout the entire pressure range.

### 2.3 Enhancement of *I*_c, sf_ on Approaching p*

We now address the most notable feature of our dataset, namely the drastic pressure dependence of *I*_c, sf_. Using the fitted *I*_c, sf_ extrapolated to 0 K as *I*_c, sf_(0), we plot its pressure dependence in Figure 4b. To facilitate the comparison of different samples, we normalize *I*_c, sf_(0) under pressure to the ambient pressure value [*I*_c, sf_(0)]_*p* = 0_ for each sample. Thus, near p*, *I*_*p*_ = [*I*_c, sf_(0)]_*p*_/[*I*_c, sf_(0)]_*p* = 0_ increases by nearly 10‐fold compared with the value at ambient pressure. To address whether the enhancement of *I*_c, sf_(0) is solely due to the increase in *T*_c_ near p*, we explored the relationship between *I*_c, sf_ and *T*_c_. As the simplest scenario, we plot the pressure dependence of *I*_*p*_ divided by *T*_c_ in Figure 4c. In the vicinity of p*, the ratio increases by ≈6‐fold. In our thin flakes, we presume *I*_c, sf_(0)∝*J*_c, sf_(0)∝λ^−3^, according to Equation (1). Assuming the applicability of Uemura relation, which states that *T*_c_∝λ^−2^,^[^ ^49^ ^]^ we then have $I_{c,\mathrm{sf}}\left(0\right)\propto T_{c}^{1.5}$. Thus, we also plot the pressure dependence of $I_{p}/T_{c}^{1.5}$ (Figure 4d). As can be seen, near p*, $I_{p}/T_{c}^{1.5}$ is still markedly enhanced by ≈5‐fold over the ambient‐pressure ratio. Therefore, the enhancement in *I*_c, sf_(0) on approaching p* is genuine, and cannot be merely explained by the *T*_c_ enhancement. To best visualize the pressure dependence of *I*_c, sf_(*T*) in the context of the phase diagram, a contour map is overlaid as displayed in Figure 1c: *I*_c, sf_ is gradually enhanced on approaching p* ≈ 20 kbar.

The peaking of the critical current has been observed and discussed alongside quantum criticality in diverse systems. In heavy fermion CeRhIn_5_, a peak shows up in the pressure dependence of zero‐field *I*_c_(0) at a hidden QCP, where a continuous antiferromagnetic transition is suppressed.^[^ ^50^ ^]^ Moreover, there is a noticeable peak in *I*_c, sf_(0) at a unique doping level *x* ≈ 0.19 in the cuprate Bi_2_Sr_2_CaCu_2_O_8 + δ_.^[^ ^38^, ^51^ ^]^ In another system (Ca_*x*_Sr_1 − *x*_)_3_Rh_4_Sn_13_, when it is tuned toward the structural QCP by chemical pressure, a significant increase in *J*_c, sf_(0) has also been observed.^[^ ^40^ ^]^ Hence, based on the aforementioned evidence, the observed peak in *I*_c, sf_(0) near p* in CsV_3_Sb_5_ thin flake appears to stem from the enhanced quantum fluctuations near the edge of the charge‐ordered phase.

Recently, quantum oscillation studies straddling p* have detected an enhancement of the quasiparticle effective masses (m*) near p*, highlighting the presence of fluctuations associated with the destabilization of the charge‐ordered phase. Because λ^−2^ = ρ_s_µ_0_ *e* ^2^/m*, the enhancement of m* near p* implies a smaller λ^−2^ and a suppressed *I*_c, sf_. To reconcile with our data, the superfluid density ρ_s_ must also increase near p*, and significantly more rapidly than m*. Therefore, it is the increase of the *ratio* (ρ_s_/m*) that contributes to the peak in critical current. Indeed, the enhancement of (ρ_s_/m*) at the critical hole concentration 0.19 has long been recognized in cuprates,^[^ ^52^, ^53^, ^54^, ^55^ ^]^ and a similar behavior can be expected for CsV_3_Sb_5_. Perhaps the most puzzling case concerns BaFe_2_(As_1 − *x*_P_*x*_)_2_, in which a striking peak in λ^2^ has been observed at a QCP – the very opposite of what is reported here.^[^ ^56^ ^]^ However, *J*_c_ measured by either the magnetic hysteresis loop method^[^ ^57^, ^58^ ^]^ or by the transport method^[^ ^59^ ^]^ also demonstrates a maximum value when the system is tuned toward the QCP. Next, also for BaFe_2_(As_1 − *x*_P_*x*_)_2_, the lower critical field, which is proportional to λ^−2^, also peaks at the QCP. Returning to the present case of CsV_3_Sb_5_, in a recent nuclear quadrupole resonance (NQR) measurement, the linewidth δν of ^121/123^Sb‐NQR spectra has been shown to follow a Curie–Weiss temperature dependence.^[^ ^30^ ^]^ At ≈1.9 GPa, close to our p*, the Weiss temperature is zero which has been regarded as evidence of a CDW QCP.^[^ ^30^ ^]^ Finally, a pressure‐induced QCP around p* has been suggested by theoretical proposals.^[^ ^28^, ^29^ ^]^ Therefore, taking into account high‐pressure results from multiple probes, the pressure dependence of *I*_c, sf_ unambiguously confirms the occurrence of a quantum phase transition beneath the superconducting dome. Our work successfully demonstrates that the concept of quantum phase transition can be employed for optimizing the critical current of the kagome superconductor CsV_3_Sb_5_, and likely for many other systems as well. As an extension of the present work, the effect of chemical substitution on CsV_3_Sb_5_ should be explored. For example, Sn‐doping has been shown to give rise to a double *T*_*c*_ dome.^[^ ^60^ ^]^ A careful *I*_c, sf_ study will further shed light on the interplay between the CDW phase and superconductivity.

## 3 Conclusion

In summary, we have explored the possibility of enhancing the critical current of CsV_3_Sb_5_ by driving the system through a quantum phase transition. We have investigated two CsV_3_Sb_5_ thin flakes with thicknesses of 225 and 180 nm via electrical transport measurements, and our temperature‐pressure phase diagram reveals the rapid suppression of the CDW phase under pressure with *T*_c_ peaking near the CDW suppression pressure p* ≈ 20 kbar. Based on the temperature–pressure phase diagram, we have performed the self‐field critical current measurements spanning a wide pressure range that includes p*. The superconducting gap Δ_0_, extracted from a single *s*‐wave gap fit, exhibits non‐monotonic behavior but closely tracks the pressure dependence of *T*_c_. Notably, the critical current exhibits a substantial enhancement of at least 10‐fold near p* compared with that at ambient pressure. The fact that the critical current at the zero‐temperature limit shows a peak at p* implies the presence of a quantum phase transition associated with the fading CDW phase in CsV_3_Sb_5_, which is consistent with other experimental data based on normal state properties.^[^ ^6^, ^30^, ^31^, ^43^, ^45^ ^]^ Our findings thus highlight the possibly generic potential for increasing critical current density through the proximity of a quantum phase transition, paving the way for systematically enhancing the critical current in many other material families.

## 4 Experimental Section

### Sample Synthesis, Characterization and Exfoliation

High‐quality single crystals of CsV_3_Sb_5_ were synthesized from Cs (ingot, 99.95%), V (powder, 99.9%) and Sb (shot, 99.9999%) using self‐flux method similar to Refs. [3, 4]. The raw materials were sealed with the molar ratio of Cs:V:Sb = 7:3:14 inside a pure‐Ar‐filled stainless steel jacket. The as‐grown single crystals were millimeter‐sized shiny plates. X‐ray diffraction (XRD) experiments of single crystals were conducted at room temperature using a Rigaku x‐ray diffractometer with CuKα radiation. The chemical compositions were determined by a JEOL JSM‐7800F scanning electron microscope equipped with an Oxford energy‐dispersive x‐ray (EDX) spectrometer. Two CsV_3_Sb_5_ thin flakes with thicknesses of 225 nm (S1) and 180 nm (S2), respectively, were exfoliated using the “blue tape” (from Nitto Denko Co.) from bulk single crystals from the same batch. These flakes were then appended to the silicone elastomer polydimethylglyoxime (PDMS, Gelfilm from Gelpak) stamp, ready to be integrated into the high‐pressure diamond anvil cell.

### Diamond Anvil Cells

The “device‐integrated diamond anvil cell” (DIDAC) technique^[^ ^61^, ^62^ ^]^ was employed to conduct the electrical transport measurements of both CsV_3_Sb_5_ flakes under high pressure. The pressure cells are equipped with two diamond anvils (Type IIas) with culet diameter of 800 µm and a bevel of 1000 µm. The schematic drawing of the diamond anvil cell is shown in Figure S1a (Supporting Information). The photolithography technique and physical vapor deposition (PVD) coating were used to attach a pattern of the six‐probe Au microelectrodes on the culet of a diamond anvil. The alumina/Stycast‐1266 mixture was placed between the stainless steel gasket and the microelectrodes as the insulating layer. The exfoliated CsV_3_Sb_5_ thin flake was then transferred onto the center of the culet. After that, the CsV_3_Sb_5_ thin flake was encapsulated by a h‐BN thin film to protect the sample from oxidization. A top view of S1 positioned on the anvil culet can be seen in Figure S1b (Supporting Information). The thickness of the thin flakes was determined by a dual‐beam focused ion beam system (Scios 2 DualBeam by Thermo Scientific). Finally, glycerin with a high purity of 99.5% was used as the pressure transmitting medium and the spectrum of ruby fluorescence at room temperature was used to calibrate the pressure on the sample.

### Electrical Transport Measurements

The electrical resistance and self‐field critical current measurements were performed by a standard four‐terminal configuration in the Physical Property Measurement System (PPMS) by Quantum Design and a dilution refrigerator by Bluefors. The six‐probe Au microelectrodes mentioned above were used to build a robust electrical contact with the CsV_3_Sb_5_ thin flake. The voltage–current (*V*–*I*) curves were measured using a Keithley 2182A nanovoltmeter in conjunction with a Keithley 6221 current source operating in the pulsed delta mode. The pulsed current had a duration of 11 ms, with a pulse repetition time of 1 s.

## Conflict of Interest

The authors declare no conflict of interest.

## Supporting information

Supporting Information

## Data Availability

The data that support the findings of this study are available from the corresponding author upon reasonable request.
