# Supplementary material for: Quantum Phase Transition as a Promising Route to Enhance the Critical Current in Kagome Superconductor CsV3Sb5
Source: Adv Sci (Weinh). 2024 Oct 16;11(45):2410099. doi: 10.1002/advs.202410099 (PMC11615797; doi:10.1002/advs.202410099)
Supplement: Supplementary file 1 — Supporting Information [file ADVS-11-2410099-s001.pdf]

ADVANCED SCIENCE
Open Access

Supporting Information

for *Adv. Sci.*, DOI 10.1002/advs.202410099

Quantum Phase Transition as a Promising Route to Enhance the Critical Current in Kagome Superconductor CsV_3Sb_5

*Wenyan Wang, Lingfei Wang, Xinyou Liu, Chun Wai Tsang, Zheyu Wang, Tsz Fung Poon, Shanmin Wang, Kwing To Lai, Wei Zhang, Jeffery L. Tallon and Swee K. Goh**

Quantum Phase Transition as a Promising Route to Enhance the Critical Current in Kagome Superconductor CsV_3Sb_5

Wenyan Wang,[1] Lingfei Wang,[1] Xinyou Liu,[1] Chun Wai Tsang,[1] Zheyu Wang,[1] Tsz Fung Poon,[1] Shanmin Wang,[2] Kwing To Lai,[1] Wei Zhang,[1] Jeffery L. Tallon,[3] and Swee K. Goh[1]

[1]*Department of Physics, The Chinese University of Hong Kong, Shatin, Hong Kong, China*
[2]*Department of Physics, Southern University of Science and Technology, Shenzhen, Guangdong, China*
[3]*Robinson Institute, Victoria University of Wellington, Wellington 6140, New Zealand*
(Dated: September 25, 2024)

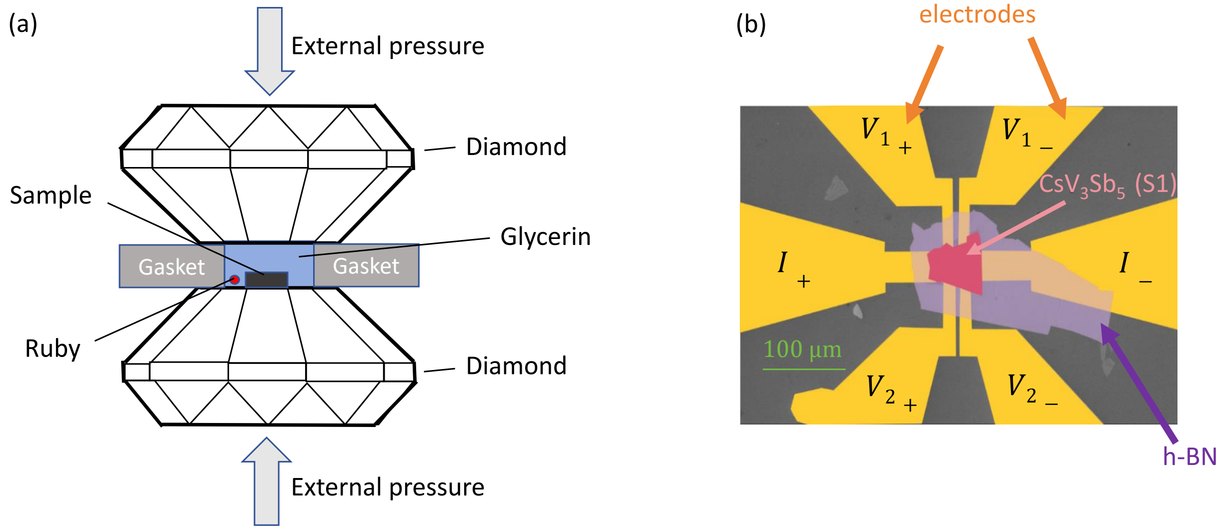

Figure S1. (a) Schematic of the diamond anvil cell. (b) Top view of a anvil culet, where the Au electrodes and the CsV_3Sb_5 thin flake can be seen. The thin flake, S1 in this instance, is covered with h-BN thin layer. False colors are used for illustration.

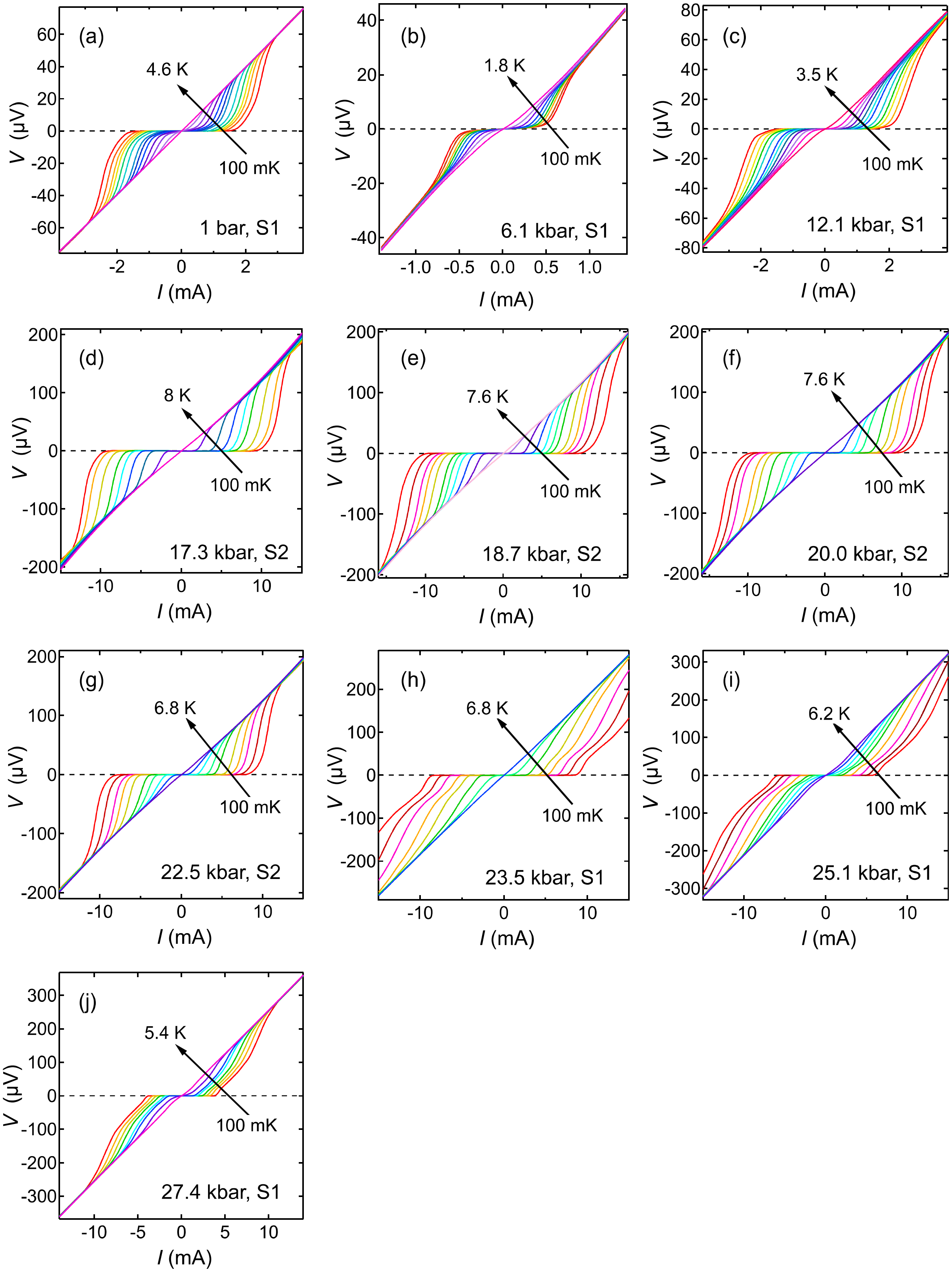

Figure S2. Temperature dependence of V-I relations at (a) ambient pressure, (b) 6.1 kbar, (c) 12.1 kbar, (d) 17.3 kbar, (e) 18.7 kbar, (f) 20.0 kbar, (g) 22.5 kbar, (h) 23.5 kbar, (i) 25.1 kbar and (j) 27.4 kbar. S1 and S2 denote the thin flakes used. The solid arrows indicate the increase of temperature from 100 mK to just above T_c.

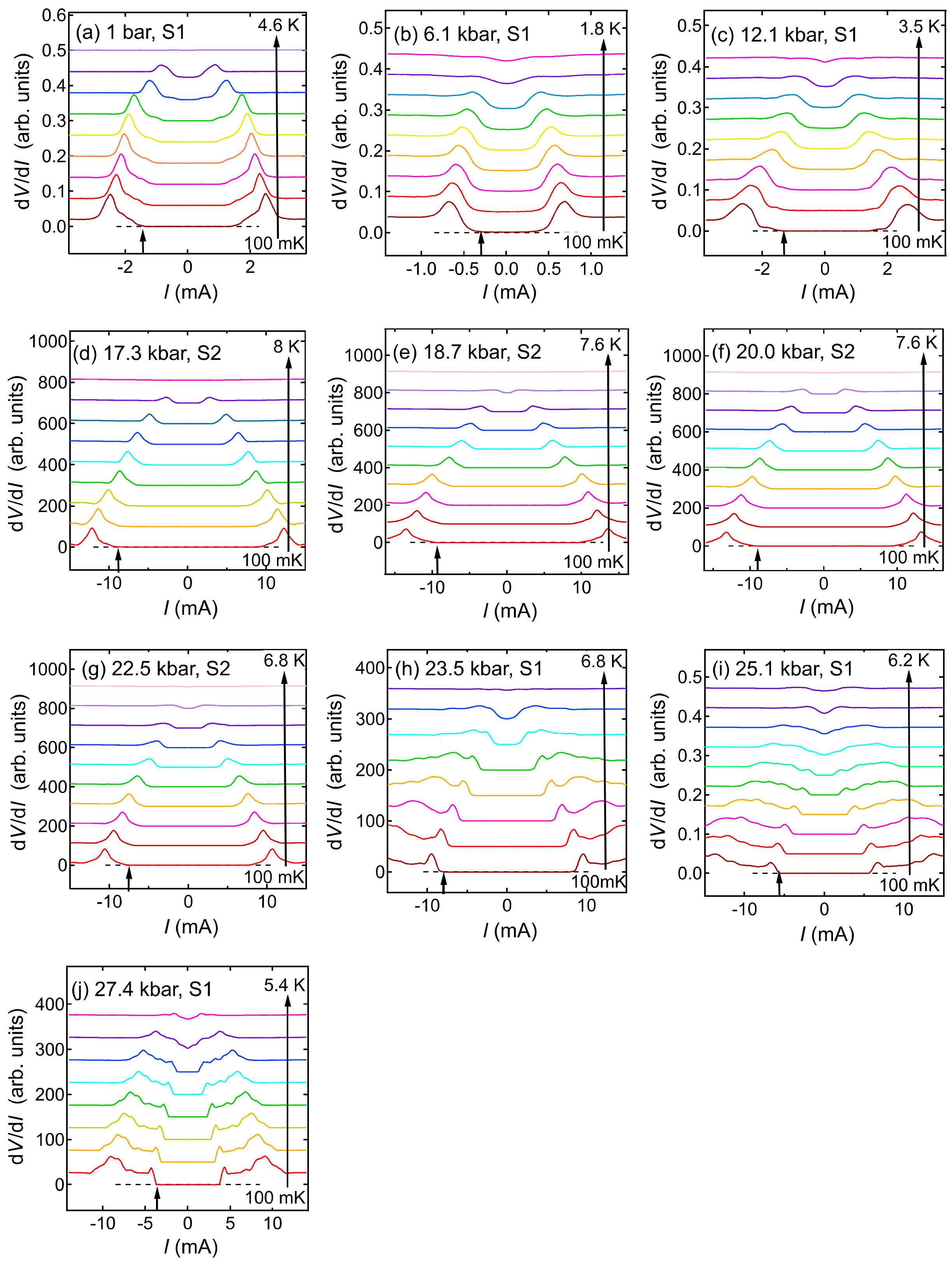

Figure S3. The calculated first derivative dV/dI at (a) ambient pressure, (b) 6.1 kbar, (c) 12.1 kbar, (d) 17.3 kbar, (e) 18.7 kbar, (f) 20.0 kbar, (g) 22.5 kbar, (h) 23.5 kbar, (i) 25.1 kbar and (j) 27.4 kbar. S1 and S2 denote the thin flakes used. The short arrows indicate the value of self-field critical current, which is defined as the current when dV/dI first deviates from zero. The long arrows demonstrate the increase of temperature from 100 mK to the temperature just above T_c.

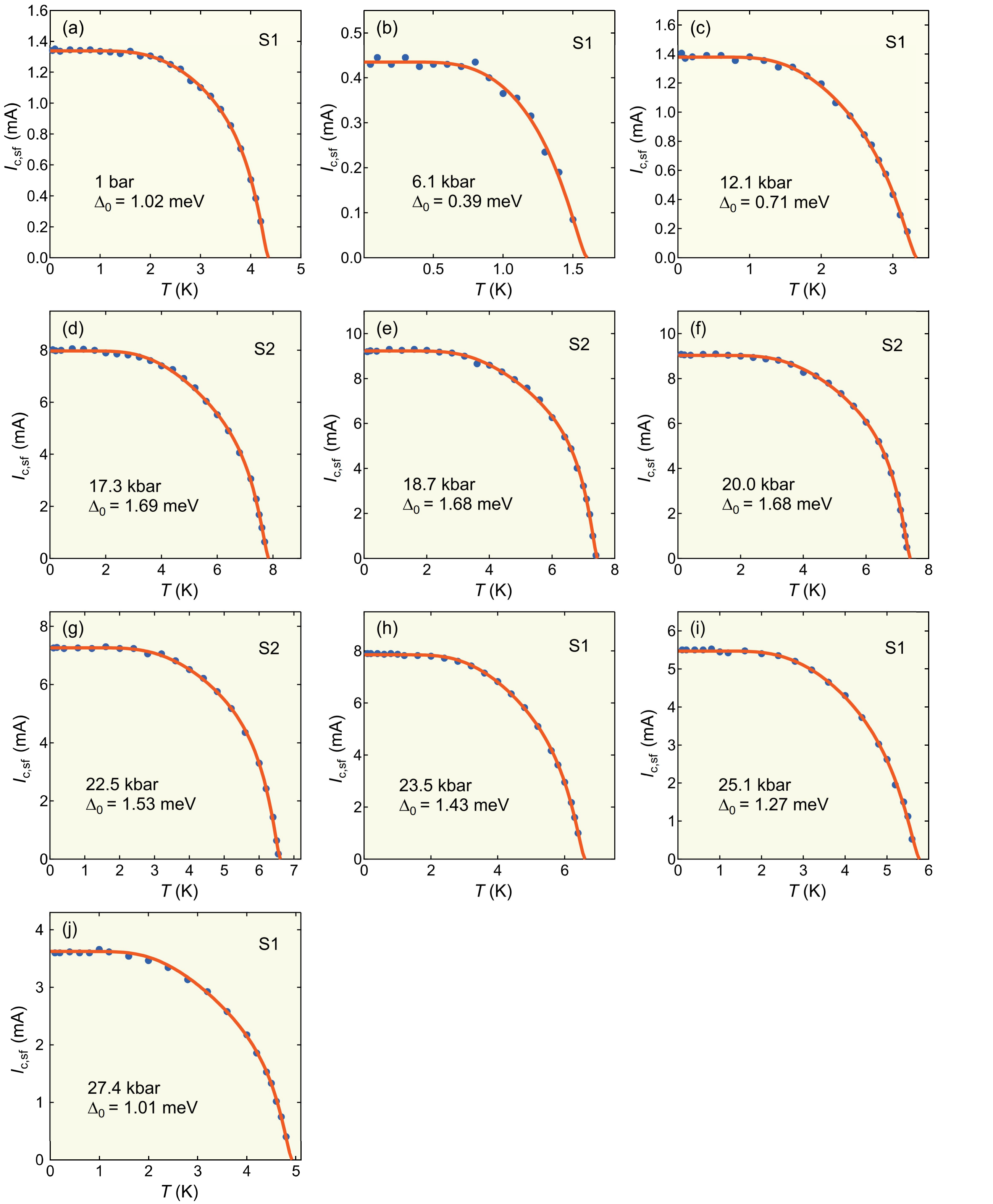

Figure S4. Temperature dependence of $I_{\mathrm{c,sf}}$ at (a) ambient pressure, (b) 6.1 kbar, (c) 12.1 kbar, (d) 17.3 kbar, (e) 18.7 kbar, (f) 20.0 kbar, (g) 22.5 kbar, (h) 23.5 kbar, (i) 25.1 kbar and (j) 27.4 kbar. S1 and S2 denote the thin flakes used. The solid curves are fits using a single s-wave gap. See the description in the main text for details. The extracted superconducting gap Δ_0 are 1.02 meV at ambient pressure, 0.39 meV at 6.1 kbar, 0.71 meV at 12.1 kbar, 1.69 meV at 17.3 kbar, 1.68 meV at 18.7 kbar, 1.68 meV at 20.0 kbar, 1.53 meV at 22.5 kbar, 1.43 meV at 23.5 kbar, 1.27 meV at 25.1 kbar and 1.01 meV at 27.4 kbar.

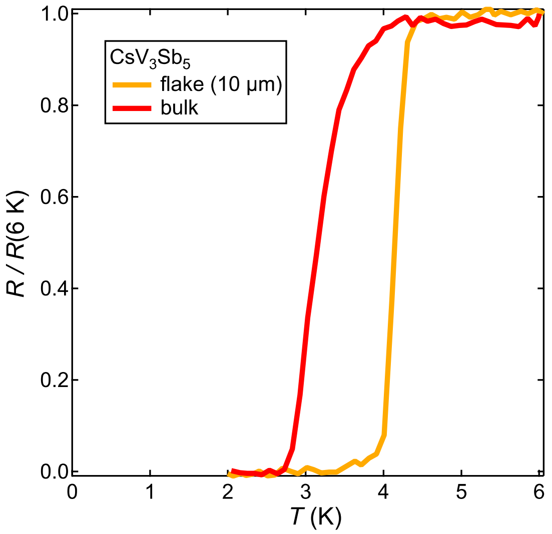

Figure S5. $R(T)$ normalized to $R(6\ \mathrm{K})$ for a biaxial-strained *thick* CsV_3Sb_5 flake (10 μm) placed on a diamond anvil (yellow trace, T_c = 3.9 K) and a free-standing bulk CsV_3Sb_5 (red trace, T_c = 2.7 K). T_c of the bulk sample is consistent with Refs. [S1, S2]. Both datasets were collected at CUHK. Compared with the bulk CsV_3Sb_5, the *thick* CsV_3Sb_5 flake has a sharper transition and higher T_c due to the biaxial strain effect from thermal expansion mismatch at the sample/diamond interface (see Refs. [S3, S4] for details of biaxial strain effect). Since strain can be effectively transmitted from the sample/diamond interface through a 10-μm-thick flake, it can also act effectively on our 200-nm-thick flakes (T_c = 4.3 K).

[S1] F. Yu, D. Ma, W. Zhuo, S. Liu, X. Wen, B. Lei, J. Ying, and X. Chen, "Unusual competition of superconductivity and charge-density-wave state in a compressed topological kagome metal," Nat. Commun. **12**, 3645 (2021).

[S2] K. Y. Chen, N. N. Wang, Q. W. Yin, Y. H. Gu, K. Jiang, Z. J. Tu, C. S. Gong, Y. Uwatoko, J. P. Sun, H. C. Lei, J. P. Hu, and J.-G. Cheng, "Double Superconducting Dome and Triple Enhancement of T_c in the Kagome Superconductor CsV_3Sb_5 under High Pressure," Phys. Rev. Lett. **126**, 247001 (2021).

[S3] K. Y. Yip, L. Wang, T. F. Poon, K. H. Yu, S. T. Lam, K. T. Lai, J. Singleton, F. F. Balakirev, and S. K. Goh, "Shubnikov–de Haas oscillations of biaxial-strain-tuned superconductors in pulsed magnetic field up to 60 T," APL Mater. **12** (2024).

[S4] K. Y. Yip, S. T. Lam, K. H. Yu, W. S. Chow, J. Zeng, K. T. Lai, and S. K. Goh, "Drastic enhancement of the superconducting temperature in type-II Weyl semimetal candidate $MoTe_2$ via biaxial strain," APL Mater. **11** (2023).
